# Biochemical and Functional Studies on the *Burkholderia cepacia* Complex *bceN* Gene, Encoding a GDP-D-Mannose 4,6-Dehydratase

**DOI:** 10.1371/journal.pone.0056902

**Published:** 2013-02-27

**Authors:** Sílvia A. Sousa, Joana R. Feliciano, Pedro F. Pinheiro, Jorge H. Leitão

**Affiliations:** 1 Institute for Biotechnology and Bioengineering, Centre for Biological and Chemical Engineering, Instituto Superior Técnico, Lisboa, Portugal; 2 Department of Bioenginneering, Instituto Superior Técnico, Universidade Técnica de Lisboa, Lisboa, Portugal; Universidad Nacional de La Plata, Argentina

## Abstract

This work reports the biochemical and functional analysis of the *Burkholderia cenocepacia* J2315 *bceN* gene, encoding a protein with GDP-D-mannose 4,6-dehydratase enzyme activity (E.C.4.2.1.47). Data presented indicate that the protein is active when in the tetrameric form, catalyzing the conversion of GDP-D-mannose into GDP-4-keto-6-deoxy-D-mannose. This sugar nucleotide is the intermediary necessary for the biosynthesis of GDP-D-rhamnose, one of the sugar residues of cepacian, the major exopolysaccharide produced by environmental and human, animal and plant pathogenic isolates of the *Burkholderia cepacia* complex species. V_max_ and K_m_ values of 1.5±0.2 µmol.min^−1^.mg^−1^ and 1024±123 µM, respectively, were obtained from the kinetic characterization of the *B. cenocepacia* J2315 BceN protein by NMR spectroscopy, at 25°C and in the presence of 1 mol MgCl_2_ per mol of protein. The enzyme activity was strongly inhibited by the substrate, with an estimated K_i_ of 2913±350 µM. The lack of a functional *bceN* gene in a mutant derived from *B. cepacia* IST408 slightly reduced cepacian production. However, in the *B. multivorans* ATCC17616 with *bceN* as the single gene in its genome with predicted GMD activity, a *bceN* mutant did not produce cepacian, indicating that this gene product is required for cepacian biosynthesis.

## Introduction

The rare 6-deoxysugar D-rhamnose is often a component of bacterial cell surface glycans, such as lipopolysaccharides (LPS), extracellular polysaccharides (EPS), and other glycoconjugate-containing bacterial cell components [1]–[4]. So far, and with the exception of *Paramecium bursaria* chlorella virus 1 where D-rhamnose is believed to be a component of the major viral capsid glycoprotein, the occurrence of this rare deoxysugar seems to be restricted to bacteria [5]. This is the case of the opportunistic pathogens of the *Burkholderia cepacia* complex (Bcc), a group of 17 closely related species that can cause infections in several hosts, including the human host [6]. These bacteria emerged in the 1980s as important pathogens, in particular among cystic fibrosis patients. In Bcc bacteria, D-rhamnose is a component of the LPS [7] and of the major extracellular polysaccharide cepacian, produced by a large percentage of clinical isolates [8], [9].

Cepacian has been pointed out as contributing to the overall pathogenicity of Bcc bacteria. For example, cepacian interferes with human neutrophils phagocytosis by facilitating the bacterial persistence in a mice model of infection, inhibits the production of oxygen reaction species (ROS) by neutrophils, and scavenges ROS, playing a role in the survival of cepacian-producing strains in different environments [10]–[14]. In addition, mutants defective in cepacian production were found as less virulent or completely avirulent in a mice model of infection [15].

Besides D-rhamnose, cepacian also contains residues of D-glucose, D-mannose, D-galactose, and D-glucuronic acid as sugar components of its heptasaccharide repeat unit [16].

Cepacian was previously identified as the sole EPS produced by the clinical isolate *B. cepacia* IST408 [8]. In addition, FTIR analysis of the EPSs produced by *B. cepacia* IST408 and *B. multivorans* ATCC17616 showed that both strains produced EPSs with FTIR spectra identical to that of cepacian [17].

Previous work allowed the identification of genes involved in cepacian biosynthesis in two clusters, named *bceI* and *bceII* [17], [18]. Cepacian gene clusters are widespread within the genomes of all strains of the *Burkholderia* genus sequenced so far, except of *B. mallei* strains [17]. The *bceII* gene cluster comprises several genes putatively involved in the synthesis of cepacian, including the *bceM* and *bceN* genes, presumably encoding proteins with putative GDP-4-keto-6-deoxy-D-mannose reductase (RMD) and GDP-D-mannose-4,6-dehydratase (GMD) activities, respectively [17].

In the present work, we report the cloning, purification, and kinetic characterization of the *bceN* gene product with predicted GMD activity, from the highly epidemic clinical isolate *B. cenocepacia* J2315, a member of the ET12 lineage that caused several fatalities among CF patients from both sides of the Atlantic (reviewed in [6]). Our results, obtained using NMR spectroscopy, confirm that the protein has GDP-D-mannose dehydratase activity (EC.4.2.1.47), catalysing the conversion of GDP-D-mannose into the labile product GDP-4-keto-6-deoxy-D-mannose. The *B. cenocepacia* J2315 GMD protein was found to be active only when in the tetrameric form, requiring the presence of magnesium ions for activity. GMD activity was detected in the absence of NAD(P), suggesting that the cofactor is tightly bound to the enzyme.

The comparison of cepacian production ability of wild-type and mutant strains derived from the cepacian producers *B. cepacia* IST408 and *B. multivorans* ATCC17616 confirmed the requirement of this enzyme activity for its synthesis.

## Materials and Methods

### Bacterial strains, plasmids and culture conditions

Bacterial strains and plasmids used in this study are described in Table S1. When in use, Bcc strains were maintained on PIA (Pseudomonas Isolation Agar, BD) plates, supplemented with 150 µg/ml trimethoprim or 600 µg/ml kanamycin in the case of mutant strains. *Escherichia coli* strains were maintained on Lennox broth (LB; containing in g/l, tryptone 10, yeast extract 5, NaCl 5) agar plates, supplemented with 100 µg/ml trimethoprim, 150 µg/ml ampicillin, or 50 µg/ml kanamycin, when appropriate. Unless otherwise stated, liquid cultures were carried out in LB liquid medium supplemented with the appropriate antibiotics with orbital agitation (250 rpm), at 30°C for Bcc strains or 37°C for *E. coli* strains. Bacterial growth was assessed by measuring the cultures optical density at 640 nm (OD_640_).

### Molecular biology techniques

Genomic DNA was extracted from exponentially-growing liquid cultures of Bcc strains using the High Pure PCR Template Preparation Kit (Roche). Plasmid isolation and purification, DNA amplification, restriction and ligation were carried out, respectively, using kits from Macherey-Nagel, and enzymes from Finnzymes and Fermentas. Agarose gel electrophoresis and *E. coli* transformation were carried out using standard procedures [19]. Electrotransformation of Bcc strains was performed as previously described [20]. Amplification of the *B. cenocepacia* J2315 *bceN* gene (BCAM1004) was performed using the oligonucleotides UP-GMD (5′-TTGCTAGCATGAGCCAAA CTC-3′) and LW-GMD (5′-AAAAAGCTTGAACGTGTCATG-3′) containing, respectively, the NheI and HindIII restriction sites (underlined) at their 5′ ends. For the amplification of the *bceN* gene and the neighbour region, oligonucleotides UP-GMDmut (5′-AAAGGTACCGTCGGAGAAATC-3′) and LW-GMDmut (5′-TTTAAGCTTC GATTCGTTCTG-3′) containing at their 5′ end, respectively, the KpnI and HindIII restriction sites (underlined). These primers were designed based on the genome sequence of *B. cenocepacia* J2315, available at the Sanger Institute Homepage (http://www.sanger.ac.uk/Projects/ B_cenocepacia).

### Nucleotide and amino acid sequence analysis and structure prediction

Nucleotide and amino acid sequences were analysed using bioinformatic tools resident at the National Center for Biotechnology Information (NCBI) or the ExPASy-Prosite websites. Searches for homology within the genome sequences of *B. cenocepacia* J2315 and other *Burkholderia* strains were carried out using the Integrated Microbial Genomes system [21]. Protein secondary and tertiary structure predictions were performed using the Protein Structure Prediction Server (PSIPRED) [22] and I-Tasser [23], respectively. Graphics were generated using the RasWin Molecular Graphics (Windows Version 2.7.5). The phylogenetic tree of BceN homologues was constructed with CLUSTAL X2, using the neighbour-joining method with a minimum of 100 bootstraps [24].

### Cloning and overexpression of *B. cenocepacia* J2315 6×His-tagged BceN

The *B. cenocepacia* J2315 *bceN* gene sequence (BCAM1004) lacking the stop codon was amplified using *B. cenocepacia* J2315 genomic DNA as template and primers UP-GMD and LW-GMD. The 1061 bp amplicon was digested with NheI and HindIII and was subsequently cloned into the NheI/HindIII cloning sites of pET23a+, yielding plasmid pJFR4. pJFR4 allows the control of protein expression by the T7 promoter, expressing the BceN protein with a 6×His-tag at its C-terminus. Plasmid pJFR4 was sequenced to confirm the correct insertion of the *bceN* gene, and transformed into *E. coli* BL21 (DE3). Overexpression of the C-terminally 6×His-tagged BceN protein was performed by growing *E. coli* BL21 (pJFR4) in 100 ml of LB liquid medium (supplemented with 150 µg/ml ampicillin) at 37°C and with orbital agitation (250 rpm). When the culture OD_640_ reached 0.6, isopropylthiogalactoside (IPTG) was added (final concentration 0.4 mM) and incubation was prolonged for 2 hours. Bacteria were harvested by centrifugation at 7000× *g* for 5 min at 4°C, and ressuspended in 8 ml of start buffer (20 mM sodium phosphate, 500 mM NaCl, pH 7.4) containing 20 mM imidazole. Cell suspensions were kept at −80°C until further processing. Aliquots of these cell suspensions were processed and protein overproduction was assessed by 12.5% sodium dodecyl sulfate polyacrylamide gel electrophoresis (SDS-PAGE). To confirm the overproduction of the 6×His-tagged BceN protein, the samples were transferred onto a nitrocellulose membrane. The membrane was incubated with a monoclonal anti-polyhistidine antibody conjugated with peroxidase (Sigma-Aldrich) diluted 1∶2000, followed by the addition of the peroxidase substrate 3,3′,5,5′ tetramethylbenzidine (TMB, Sigma).

### Purification of the 6×His-tagged BceN

Cell suspensions prepared as described above were lysed by ultrasonic vibration with a Branson sonifier 250 (Branson). Cell debris were removed by centrifugation at 12,500× *g* for 1 hour at 4°C. The 6×His-tagged BceN protein was purified by affinity chromatography using a Hi-Trap chelating column (GE Healthcare), following the suppliers instructions. Purified fractions were analysed by SDS-PAGE followed by staining with Coomassie brilliant blue R-250. The eluted fractions containing the purified 6×His-tagged protein were dialysed overnight at 4°C in a 10 kDa cutoff Slide-A-Lyzer Dialysis Cassette (Pierce) against 25 mM sodium phosphate buffer (pH 7.4) supplemented with 50 mM NaCl and 20% (v/v) glycerol. Protein concentration was estimated by the method of Bradford [25], with bovine serum albumin fraction V (Nzytech) as standard.

### Discontinuous native protein gel electrophoresis

Native protein gel electrophoresis of the purified 6×His-tagged BceN was performed based on previously described methods [26] with minor modifications to estimate the molecular mass of the 6×His-tagged BceN oligomer. Briefly, 20 µl aliquots of protein samples (6 µg) were added to 5 µl of loading buffer (100 mM Tris-Cl pH 8.0, 40% glycerol, 0.5% Brilliant Blue G) and incubated for 10 minutes at room temperature. Samples containing 20 µg of catalase (232 kDa), aldolase (157 kDa), bovine serum albumin (66 and 132 kDa), and ovalbumin (44 kDa) were used as molecular mass standards. The samples were applied to an 8% (wt/vol) polyacrylamide gel containing 200 mM Tris-Cl (pH 8.8). The cathode buffer was composed of 100 mM histidine adjusted to pH 8.0 using Tris base (without chloride) and 0.002% Brilliant Blue G. The anode buffer contained 100 mM Tris-Cl (pH 8.8). The gel was run at 4°C and 100 V for 2 hours.

### Assessment of GDP-D-mannose 4,6-dehydratase (GMD) activity by NMR

The ability of the *B. cenocepacia* J2315 6×His-tagged BceN protein to convert GDP-D-mannose into GDP-4-keto-6-deoxy-D-mannose was assessed using GDP-D-mannose as substrate, and analysing the reaction products by Nuclear Magnetic Resonance (NMR) spectroscopy, based on the methods described by King *et al.* [5]. NMR experiments were performed using a Bruker AVANCE II^+^ 400 MHz (^1^H) spectrometer equipped with a 5 mm BBO probe. The reacting mixture [containing 25 mM sodium phosphate buffer, 50 mM NaCl, 20% (v/v) glycerol, 90% H_2_O/10% D_2_O, pH 7.4, in a total volume of 500 µl], was placed in a 5 mm NMR tube (Norrel 100 MHz) with concentrations of GDP-D-mannose (Sigma) ranging from 1 to 5 mM, in the absence or presence of 5 mM NADP^+^ (Sigma), and MgCl_2_ (1 mol MgCl_2_ per 1 mol of purified 6×His-tagged BceN protein). Experiments were conducted at 25°C. The reaction was started by the addition of 90 µg of the purified 6×His-tagged BceN protein to the reaction buffer, and the proton spectrum was recorded at fixed periods of time. Spectral data was collected over time and treated with TopSpin software v2.1. Resonance of the acetone methyl group was used as internal reference for all spectra (CH_3_ **δ**_H_ = 2.225 ppm, CH_3_ **δ**_C_ = 30.89 ppm). Different concentrations of MgCl_2_ (0 mM, 25 mM and 1 mol∶1 mol protein) and NADP^+^ (0 or 5 mM; Sigma), and different reaction temperatures (25°C and 37°C) were tested for the reaction conditions optimization. Reaction products were characterized by 1D (^1^H and ^13^C) and 2D (HSQC) NMR experiments. Protein stability was only achieved when using 20% (v/v) glycerol in all experiments, which interfered with the substrate and product peaks in the range of 3.5 to 3.9 ppm. Therefore, kinetic measurements were based on the proton signals corresponding to the anomeric protons in GDP-D-mannose and the two reaction products as described by King and coworkers [5]. Integral values of the 5.51 ppm signal were normalized with the internal standard (CH_3_ **δ**_H_ = 2.225 ppm) and plotted versus time [27]. V_o_ was obtained from the slope of the exponential part of the curve obtained with the increase of products concentration, calculated using the equation [P]_t_ = [S] (1-SNA_t_), where [P]_t_ is the concentration of products on time t, [S] is the initial substrate concentration, and SNA_t_ is the substrate normalized integral signal area at time t. NMR experimental data was further analyzed using Graphpad Prism v6.

The hypothesis that the 6×His-tagged BceN protein can catalyse both the GDP-D-mannose 4,6-dehydratation and the subsequent reduction reaction to produce GDP-D-rhamnose was investigated based on the two following NMR assays: a) addition of 10 mM NADPH to the reaction mixture [containing 90 µg of the purified BceN protein, 5 mM GDP-D-mannose, MgCl_2_ at the ratio of 1 mol MgCl_2_ per 1 mol protein, 25 mM sodium phosphate buffer, 50 mM NaCl, 20% (v/v) glycerol, 90% H_2_O/10% D_2_O, pH 7.4]; b) addition of 10 mM NADPH, after incubation of the reaction mixture for 30 minutes at 25°C.

### Generation of mutants and PCR confirmation of insertional inactivation

The *bceN* (BCAM1004) gene and the neighbour region was amplified from *B. cenocepacia* J2315 genomic DNA using primers UP-GMDmut and LW-GMDmut. The 2272 pb amplimer was digested with KpnI and HindIII and subsequently cloned into the KpnI/HindIII cloning sites of pDrive, generating pACM2. The 911 bp EcoRI/SphI fragment from pUC-Tp containing the Tp cassette was ligated into the EcoRI/SphI sites within the *bceN* gene cloned in pACM2, creating pACM4. This last plasmid was introduced in *E. coli* SSC110 and extracted from this strain to obtain non-methylated plasmid DNA for transformations, to escape the Bcc restriction system [28]. The extracted plasmid was then introduced into *B. cepacia* IST408 and *B. multivorans* ATCC17616 by electrotransformation, as previously described [20]. In order to confirm the insertional inactivation of the *bceN* gene and the double cross-over recombination (expected size of 3183 bp), colony-PCR experiments were performed, using the primers UP-GMDmut and LW-GMDmut.

The 1061 bp XbaI/HindIII fragment from pJRF4 containing the *bceN* gene was subcloned into the XbaI/HindIII restriction sites of pDrive, creating pJFR5. The 1125 bp KpnI/XbaI fragment from pJFR5 was then inserted into pMLBAD, yielding pJRF6. The resulting plasmid (pJRF6) expresses an antisense RNA, under the control of the P*_BAD_* promoter, which is complementary to the *bceN* mRNA, thus allowing the silencing of *bceN* upon induction with arabinose. The plasmid was introduced into *B. multivorans* ATCC17616 by triparental conjugation as previously described [17].

### Quantification of exopolysaccharide production

EPS production was quantified by determining the dry weight of the ethanol-precipitated polysaccharide present in 30 ml of cell-free culture samples of the Bcc strains as described before [20]. The culture samples were obtained from Bcc cultures grown at 30°C or 37°C in S_ARA_ liquid medium (containing, in g/l, arabinose 20, Na_2_HPO_4_. 2H_2_O 12.5, KH_2_PO_4_ 3, K_2_SO_4_ 1, NaCl 1, yeast extract 1, casamino acids 1, MgSO_4_. 7H_2_O 0.2, CaCl_2_. 2H_2_O 0.01, FeSO_4_. 7H_2_O 0.001), for 24, 48, and 72 hours, with orbital agitation (250 rpm), as described previously [8]. EPS production was quantified as EPS dry weight (g) per litre of growth medium, and values given are the mean values of at least three independent determinations.

### Statistical analysis

The data obtained from the quantification of the ability of the *Burkholderia* strains and their respective mutant strains to produce EPS was tested for statistical significance by calculating *P*-values using the Unpaired t test with Welch correction. A *P*-value<0.05 was considered statistically significant. Statistical analyses were done with GraphPad InStat v3.

### Nucleotide sequence accession number

The *bceN* gene nucleotide sequence of *B. cepacia* IST408 was deposited in GenBank under the Accession No. JN987864.

## Results

### Sequence analysis of *B. cenocepacia* J2315 *bceN*

**Figure 1:**
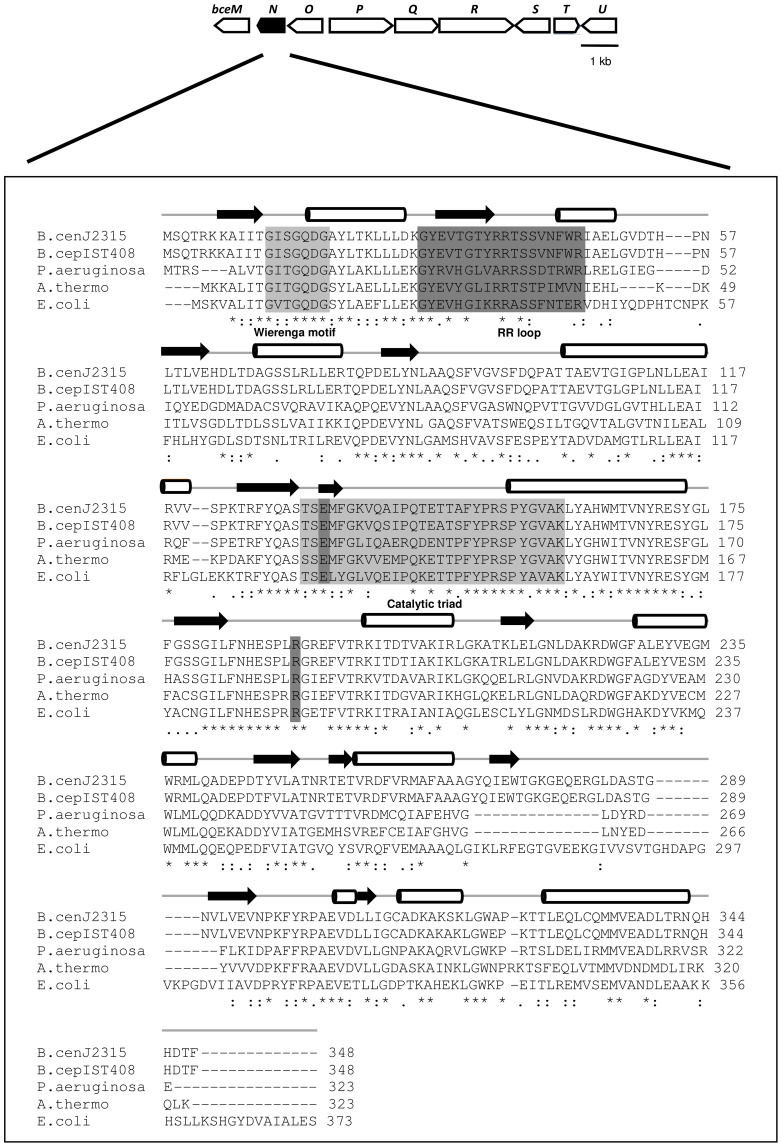
Genetic organization of the *Burkholderia cenocepacia* J2315 *bce-II* gene cluster, and alignment of amino acid sequences of the *B. cenocepacia* J2315 BceN protein (CAR54861) with GMDs from *B. cepacia* IST408 (JN987864), *P. aeruginosa* (AAG08838), *A. thermoaerophilus* (AAS55711) and *E. coli* (AAC77842). Upper part: Schematic representation of the *bceII* gene cluster evidencing the *bceN* gene in black. Lower part: Sequence alignment of the indicated GMDs, evidencing the Wierenga motif and the catalytic triad typical of the short-chain dehydrogenases/reductases (SDR) family of proteins (highlighted in light grey). The specific GMD motifs are highlighted in dark grey. Asterisks indicate identical amino acid residues. One or two dots indicate semi-conserved or conserved substitutions, respectively. The predicted secondary structure of *B. cenocepacia* J2315 and *B. cepacia* IST408 BceN proteins is shown above the alignment segments, where cylinders represent α-helices and arrows represent β-sheets. Alignments and the secondary structure predictions were performed with CLUSTAL X2 and the PSIPRED software, respectively. Genes *bceM* to *bceU* putatively encode GDP-D-rhamnose reductase (*bceM*), GDP-D-mannose dehydratase (*bceN*), acyltransferase (*bceO*), unknown (*bceP*), repeat unit flippase (*bceQ*), glycosyltransferase (*bceR*), acyltransferase (*bceS*), UDP-glucose pyrophosphorylase (*bceT*), acyltransferase (*bceU*).

The *B. cenocepacia* J2315 *bceN* gene is located in the *bce-II* gene cluster of chromosome 2, spanning nucleotides 1112176 to 1113222 (Fig. 1). The *bce-II* cluster was previously identified as being involved in the biosynthesis of the exopolysaccharide cepacian [17]. The translated sequence of *bceN* (1061 bp) was found to have a similarity higher than 66% with proteins with GMD activity from other bacterial species such as *B. cepacia*, *Pseudomonas aeruginosa*, *Aneurinibacillus thermoaerophilus*, and *E. coli* (Fig. 1). A search for BceN homologues within available genome sequences of *Burkholderia* strains allowed the identification of 23 BceN orthologues in 14 strains from 9 *Burkholderia* species. One, two or three putative GMDs were found in the genomes sequences examined. The GMD-encoding genes with highest degree of identity/similarity with BceN all belong to gene clusters similar to the *bce-II* gene cluster (Fig. S1, left upper panel) that is involved in cepacian production [Clade A in Fig. S1 panel B; 17]. The BceN homologues ABF75312, ABK07633, EAY62635 and EAY62633, EAY69559, ACB63266, ABI86318 and ABC38232 identified in the genomes of *B. cenocepacia* AU1054, *B. cenocepacia* HI2424, *B. cenocepacia* PC184, *B. dolosa* AU0158, *B. ambifaria* MC40-6, *B. ambifaria* AMMD and *B. thailandensis* E264, respectively, are all located on chromosome 1, within clusters of genes encoding putative glycosyltransferases and other proteins and enzymes presumably involved in lipopolysaccharide synthesis (Fig. S1, right upper panel). These proteins share an identity higher than 80% to each other, forming a separate clade (Clade B in Fig. S1 panel B). A GMD-encoding gene located on chromosome 3 was identified in *B. vietnamiensis* G4 (protein ABO59420) (Fig. S1 panel B). In this case, the encoding gene is flanked by proteins putatively involved in polysaccharide and lipopolysaccharide biosynthesis, and exopolysaccharide transport. This was the only sequenced strain of the *Burkholderia* genus putatively encoding on chromosome 3 a protein with GMD enzyme activity.

GMDs are members of the NDP-sugar modifying subfamily of the short-chain dehydrogenases/reductases (SDR) family [29]. These proteins catalyse the irreversible conversion of GDP-D-mannose into the unstable compound GDP-4-keto-6-deoxy-D-mannose [30], which can be further converted into GDP-D-rhamnose, GDP-L-fucose, GDP-6-deoxy-D-talose, or GDP-D-perosamine [31].

Analysis of the amino acid sequences of the translated *bceN* gene of the cepacian high producer clinical isolate *B. cepacia* IST408 and comparison with the *bceN* sequence of *B. cenocepacia* J2315 revealed that these sequences share 97% identity and 99% similarity (Fig. 1). Both proteins have a predicted molecular mass of approximately 39 kDa and a pI of 6.35. The analysis of the deduced amino acid sequences of the BceN proteins from *B. cenocepacia* J2315 and *B. cepacia* IST408 also revealed the presence in both proteins of six conserved domains typical of GMD enzymes (Fig. 1), namely: (i) the glycine-rich Wierenga motif, ^12^GXXGXXG^18^, a conserved feature of proteins of the SDR family [32], present in the BceN N-terminal domain and thought to be involved in the binding of the cofactor NAD(P)^+^; (ii) the ^34^GXXRR^38^ motif, conserved in enzymes with GMD activity, with the two positively charged arginine residues at the beginning of the RR loop [31]; (iii) the RR loop, spanning amino acid residues Arg37 to Arg46, thought to be involved in protein-protein interactions and protein-cofactor interactions to the neighbouring monomer, proposed as essential for the typical multimeric structure of GMD proteins [31]; (iv) the conserved catalytic triad S/T^131^ and ^155^YXXXK^159^, essential for catalysis. The tyrosine residue is responsible for the deprotonation of the C4 hydroxyl group of the sugar moiety, while the lysine residue is proposed to lower the pK_a_ of the tyrosine to enable the catalysis reaction [29], [30]; (v) the glutamic acid residue at position 133 that is conserved in dehydratases and play a role in the deprotonation/reprotonation of the C5 during catalysis, acting as an additional active-site base that abstracts the C5 proton in the dehydration reaction [30]; (vi) the conserved arginine residue in position 190 that is fundamental for the correct orientation of the substrate GDP-D-mannose and the NADP^+^ cofactor in the active site for the dehydratase reaction [5].

The secondary structures of BceN proteins from both *B. cenocepacia* J2315 and *B. cepacia* IST408 were predicted by PSIPRED. The predicted structures exhibit the N-terminal Rossman fold domain, typical of the sugar nucleotide-modifying SDR family (Fig. 1). This motif is composed of an α/β folding pattern, with 6–7 β-strands flanked by 3–4 α-helices on each side, proposed to be involved in cofactor binding [29].

### The *B. cenocepacia* J2315 BceN forms tetramers in its active form

In order to assess the predicted GMD activity of the *B. cenocepacia* BceN protein, the *bceN* gene was cloned in the expression plasmid pET23a+, as described in the Materials and Methods section. The resulting plasmid allows the expression of the BceN protein with a 6×His-tag at its C-terminus. The C-terminus location of the 6×His-tag was selected to prevent a possible enzymatic inactivation of BceN, which was previously reported for the *E. coli* K12 GMD enzyme, due to structural changes in the dinucleotide binding domain of the N-terminal region [33].

**Figure 2:**
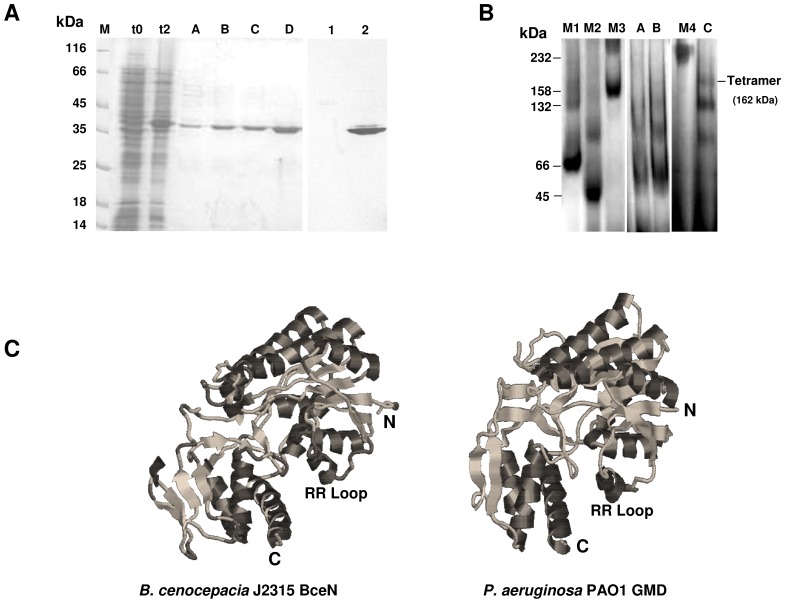
BceN is a tetramer. (**A**) SDS-PAGE analysis of the 6×His-tagged BceN from *B. cenocepacia* J2315 by *E. coli* BL21 (DE3). Lanes from left panel: t0, total soluble proteins from *E. coli* BL21 (DE3) with plasmid pJRF4 before induction with IPTG; t2, total soluble proteins from *E. coli* BL21 (DE3) with plasmid pJRF4 after 2 hours of induction with IPTG; A–D, 6×His-tagged BceN protein eluted from Ni^2+^-NTA affinity chromatography column with increasing concentrations of imidazole. Lanes from right panel (Western-blot): 1, BSA used as negative control; 2, purified 6×His-tagged BceN protein from *B. cenocepacia* J2315. The monoclonal anti-polyhistidine antibody conjugated with peroxidase (Sigma-Aldrich) was used in the Western-blot. (**B**) Discontinuous native PAGE analysis of 6×His-tagged BceN. Lanes: M1, BSA; M2, ovalbumin; M3, aldolase; M4, catalase; A–B, purified 6×His-tagged BceN from *B. cenocepacia* J2315 with no GMD activity; C, purified 6×His-tagged BceN from *B. cenocepacia* J2315 with GMD activity. The analysis of the discontinuous native PAGE showed a predominant band with approximately 162 kDa compatible with a tetrameric form of the protein. Trimeric, dimeric and monomeric forms are also visible. (**C**) Structural models of the BceN of *B. cenocepacia* J2315 and GMD from *P. aeruginosa* PAO1 (PDB ID: 1RPN). Graphics were generated using the RasWin Molecular Graphics (Windows Version 2.7.5).

The ability of the purified 6×His-tagged BceN protein to form multimers was investigated using size-exclusion chromatography (data not shown). However, a very low resolution was obtained for the purified BceN protein and the protein standards, most probably due to its high content in glycerol. Therefore, we have used discontinuous native PAGE analysis to investigate the ability of BceN to form multimers. For this purpose, two aliquots from purified 6×His-tagged BceN were used, one with GMD activity and the other without GMD activity, as detected by NMR spectroscopy analysis. Several bands of higher molecular mass than that of the band that correspond to the monomeric form of BceN could be detected (Fig. 2B). However, a band with approximately 162 kDa which corresponds to four times the molecular mass of the monomeric BceN was only detected in the sample with GMD activity, suggesting that in its active form BceN is a tetramer (Fig. 2A and 2B). Interestingly, the crystal structure determination of the *P. aeruginosa* PAO1 GMD protein (PDB ID: 1RPN) revealed that the active form of this protein is a tetramer [31]. Although the *B. cenocepacia* BceN and the *P. aeruginosa* GMD share only 73% similarity (Fig. 1), the comparison of the predicted 3D structures of the BceN monomer with the *P. aeruginosa* PAO1 GMD (PDB ID: 1RPN) revealed a high level of structural similarity (Fig. 2C). One of the features observed was the conserved spatial location of the RR loop that it is thought to play a role on the interactions with the adjacent monomer in the tetrameric structures of GMD proteins, as well as with their cofactor when in the tetramer arrangement.

### BceN exhibits GDP-D-mannose 4,6 dehydratase (GMD) activity

The enzyme activity of the 6×His-tagged BceN protein of *B. cenocepacia* J2315 was investigated using the protein purified to homogeneity by affinity chromatography, followed by dialysis overnight at 4°C against dialysis buffer (25 mM sodium phosphate buffer, pH 7.4; 20% (v/v) glycerol; 50 mM NaCl). SDS-PAGE analysis of the purified 6×His-tagged BceN protein showed an apparent molecular mass of approximately 40 kDa (Fig. 2A), consistent with its predicted molecular mass of 39 kDa.

**Figure 3:**
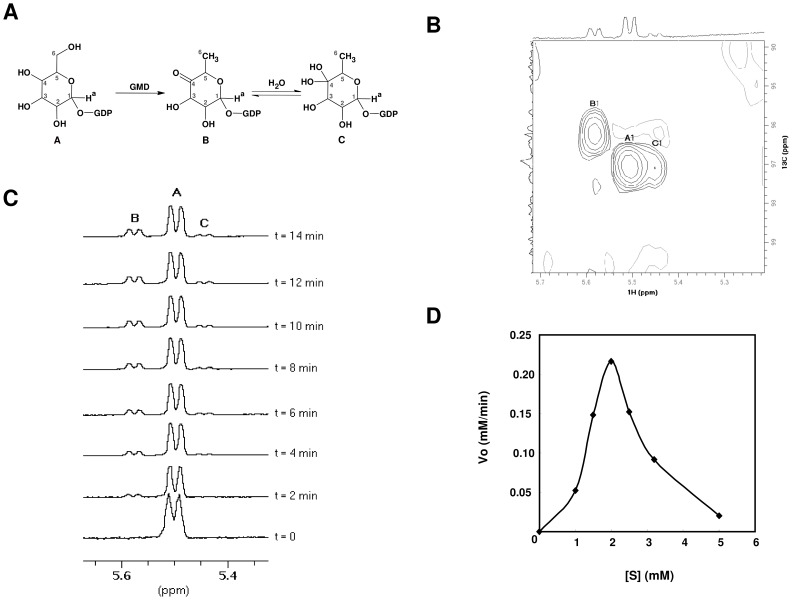
NMR spectroscopy studies of the 6×His-tagged BceN-catalysed reaction. (**A**) Conversion of GDP-D-mannose (compound A) into GDP-4-keto-6-deoxy-D-mannose keto form (compound B) and the gem-diol form of compound B (compound C). The anomeric protons in each molecule are marked as H^a^. (**B**) HSQC spectra of the reaction mixture containing 5 mM of GDP-D-mannose, after 14 hours of incubation at 25°C. The anomeric protons and corresponding signals in HSQC are showed. (**C**) Time-course spectra of the 6×His-tagged BceN reaction mixture with 2 mM of GDP-D-mannose at 25°C, showing the peaks corresponding to GDP-D-mannose (A), GDP-4-keto-6-deoxy-D-mannose keto form (B), and the gem-diol form of compound B (C). (**D**) Michaelis-Menten plot of the initial rate of GDP-D-mannose consumption as determined by NMR.

The GMD activity of the *B. cenocepacia* J2315 6×His-tagged BceN protein was assessed by analysing the reaction products by NMR spectroscopy (Fig. 3). The data obtained is summarized in Table S2. Proton and carbon chemical shifts for the reaction mixture products in the presence of GDP-D-mannose were in good agreement with the data obtained for the compounds identified for the GMD enzyme activity of *P. aeruginosa* [5]. A peak corresponding to the C4 of compound B (Fig. 3A) was observed at δ_C_ = 208.60 ppm, indicating a carbonyl group, as expected to occur in the keto form of GDP-4-keto-6-deoxy-D-mannose [5]. Although low resolution and glycerol interference hindered the detection of the chemical shift of the C4 of compound C, the H1 and H6 proton and C1 and C6 carbon chemical shifts are in accordance with the *gem*-diol form of compound B (Fig. 3A), as previously described by King and colleagues [5].

The monitorization of the anomeric region of the 1D-^1^H-spectrum showed a progressive depletion of signals corresponding to GDP-D-mannose (compound A) and an increase of the intensity of the peaks corresponding to the resonances of the 4-keto (compound B) and gem-diol (compound C) anomeric forms of GDP-4-keto-6-deoxy-D-mannose (Fig. 3B and 3C). Spectra obtained indicate that the 6×His-tagged BceN protein catalyzes the dehydration of GDP-D-mannose to GDP-4-keto-6-deoxy-D-mannose, thus having GDP-D-mannose 4,6-dehydratase activity. Results also indicate that the protein only catalyzes the dehydration reaction, since no peaks corresponding to GDP-D-rhamnose could be detected.

The requirement of a dinucleotide cofactor for the GMD activity of proteins from other bacterial species has been reported [34]. However, the GMD activity of the *B. cenocepacia* 6×His-tagged BceN protein was unaffected by the addition of exogenous NAD^+^ or NADP^+^ (data not shown). We hypothesize that the cofactor was co-purified with the protein, as previously observed for the *P. aeruginosa* GMD protein [5]. The GMD activity of the BceN protein was also found to be dependent on the addition of Mg^2+^, being the enzyme inactive in its absence (data not shown). However, high concentrations of MgCl_2_ (25 mM) had an inhibitory effect on GMD activity (data not shown). Therefore, MgCl_2_ concentration was kept constant in all experiments, at the optimized ratio of 1 mol of MgCl_2_ per 1 mol of BceN. Although the 6×His-tagged BceN protein was found as active at 37°C, its activity was lost in the time course of the enzyme-substrate incubations. In contrast, the 6×His-tagged BceN protein activity was stable at 25°C after 14 hours of incubation, similarly to the GMD protein from *P. aeruginosa* [5]. Therefore we have performed the kinetic characterization of the enzyme at 25°C.

A characteristic sigmoidal shape was observed for GDP-D-mannose concentrations up to 2 mM, being the enzyme activity low at low substrate concentrations and presenting a rapid increase in enzyme activity with increasing substrate concentrations (Fig. 3D). This observation suggests a cooperative binding of the substrate (Fig. 3D). The Hill cooperative binding model [35] was used to fit the first four points of V_o_ estimated based on the NMR experimental data using Graphpad Prism v6. This analysis revealed a Hill coefficient of 3.949, typical of a tetramer. This is consistent with the results from discontinuous native PAGE analysis, showing that the protein can exist in a tetrameric form (Fig. 2B).

A decrease in the reaction rate was observed for substrate concentrations higher than 2 mM (Fig. 3D). These results suggest that the enzyme is inhibited by the substrate. Therefore, a nonlinear least-squares algorithm was used to fit the enzyme velocity data versus the substrate concentration. The kinetic parameters were calculated using the equation *v* = *V*_max_[S]/[*K*_m_+[S] (1+[S]/*K*_i_)], which takes substrate inhibition into account. The values obtained for *K*_m_ and *V*_max_ were 1024±123 µM and 1.5±0.2 µmol min^−1^mg^−1^, respectively, corresponding to a *k*_cat_ value of 1.0 s^−1^ and *K_cat_*/*K_m_* value of 9.77×10^2^ M^−1^ s^−1^. A *K_i_* value for GDP-D-mannose of 2913±350 µM was estimated, a value almost 3-fold that estimated for *K_m_*, suggesting that the substrate binds with high affinity to the active site of the enzyme. The kinetic parameters obtained for the *B. cenocepacia* J2315 6×His-tagged BceN protein, as well as available kinetic parameters for the GMD enzymes of *P. aeruginosa*, *H. pylori*, *E. coli*, and the fungi *Mortierella alpina* are summarized in Table 1.

**Table 1:** Kinetics parameters for different bacterial GMD enzymes using GDP-D-mannose as substrate.

Protein, Bacteria	Multimeric form	pH, Temperature	K_m_ (µM)	V_max_ (µmol min^−1^ mg^−1^)	K_i_ (µM)	K_cat_ (s^−1^)	K_cat_/K_m_ (M^−1^ s^−1^)	Reference
BceN, *B. cenocepacia* J2315	Tetramer	7.4, 25°C	1024±123	1.5±0.2	2913±350	1	9.77×10^2^	This study
GMD, *P. aeruginosa*	Tetramer	7.5, 37°C	14.02±6.05	3.64±1.37	2.859±1.31	8.82	6.3×10^5^	[5]
HP0044, *H. pylori*	Tetramer	6.5, 37°C	117.3±1.38	ND	ND	0.27	2.3×10^3^	[39]
GMD, *E. coli*	Dimer	7.5, 37°C	280±12	ND	ND	5	1.8×10^5^	[30]
GMD, *Mortierella alpina*	Trimer	9.0, 37°C	770±7	ND	ND	5.96	7.74×10^3^	[44]

Some bacterial GMDs are bifunctional, being able to catalyze the 4-keto reduction of GDP-4-keto-6-deoxy-D-mannose [5]. However, our NMR assays to determine the products of BceN enzyme activity only showed peaks attributable to GDP-4-keto-6-deoxy-D-mannose as the single product of the reaction catalysed by GMD. These results indicate that the *B. cenocepacia* J2315 6×His-tagged BceN protein is a monofunctional enzyme, at least under the assay conditions used in this work.

### Construction and characterization of a *bceN* mutant

Since GDP-D-rhamnose is one of the sugar nucleotides required for the synthesis of the extracellular polysaccharide cepacian, the mutant strain *B. cepacia* JFR1 with the *bceN* gene insertionally inactivated was constructed from *B. cepacia* IST408, previously described as a high EPS producer [8]. *B. cenocepacia* J2315 was previously reported as a cepacian non-producer due to a frameshift mutation in the *bceB* gene [18]. Due to difficulties in constructing a *bceN* mutant in the EPS producer *B. multivorans* ATCC 17616, a strategy based on the interference of an antisense RNA targeting the *bceN* mRNA was employed to construct a conditional mutant. This approach has been used successfully in *Staphylococcus aureus* and Mycobacteria to analyse specific gene essentiality, and more recently in *B. cenocepacia* J2315 [36]–[38]. With this purpose, the pMLBAD vector harbouring a DNA fragment of the *bceN* gene in the antisense orientation was inserted downstream of the pBAD promoter, resulting in the antisense plasmid pJRF6. This plasmid was introduced into *B. multivorans* ATCC 17616 cells by triparental conjugation, yielding *B. multivorans* (pJRF6), which, upon induction with arabinose, expresses the antisense transcript of *bceN*, effectively blocking the translation of the mRNAs corresponding to *bceN* (results not shown).

**Figure 4:**
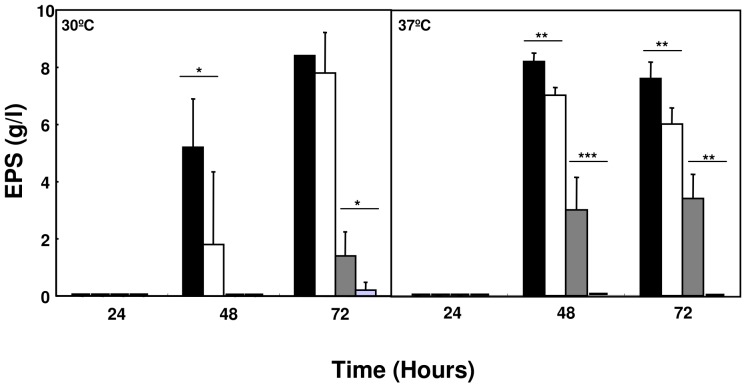
Involvement of BceN in cepacian biosynthesis. Comparison of the amount of cepacian produced by *B. cepacia* IST408 (black columns), *B. cepacia* JRF1 (white columns), *B. multivorans* ATCC17616 (dark grey columns), and *B. multivorans* (pJFR6) (light grey columns) in S_ARA_ liquid medium, after 24, 48 and 72 hours of incubation at 30°C and 37°C with orbital agitation. The P-value was determined with the Unpaired t test with Welch correction and are represented by * when P<0.01, ** when P<0.0001, *** when P<0.001.

Similar growth curves were observed for the wild-type strain *B. cepacia* IST408, the mutant *B. cepacia* JFR1, *B. multivorans* ATCC17616 and the *B. multivorans* (pJFR6) strain in S_ARA_ liquid medium (data not shown). The ability of the wild-type strain *B. cepacia* IST408, the mutant strain *B. cepacia* JFR1, *B. multivorans* ATCC17616 and *B. multivorans* (pJRF6) to produce EPS was compared in S_ARA_ liquid medium at 30°C and 37°C with orbital agitation. Although the *B. cepacia* JRF1 mutant was still able to produce EPS in S_ARA_ liquid medium, a significant reduced yield was observed when compared to the IST408 wild-type strain after 48 hours of growth (Fig. 4). Since GMD activity is required for the biosynthesis of the activated sugar precursor GDP-D-rhamnose of the cepacian EPS, we expected that the loss of GMD would have a stronger effect on EPS production. The ability to produce EPS by the *bceN* mutant strain suggested that additional genes encoding for proteins with GMD activity might be present within the genome of *B. cepacia* IST408. A similar situation was previously reported by our research group for the *bceA* gene, encoding the bifunctional protein with both phosphomannose isomerase and GDP-D-mannose pyrophosphorylase activities [20]. Results from southern hybridization experiments with total DNA from *B. cepacia* IST408 and a probe based on *bceN* gene suggest that more than one GMD-encoding gene is present in this genome, similarly to *B. cenocepacia* PC184 (results not shown). Therefore, we have conducted homology searches for putative GMD encoding genes within publicly available genome sequences of strains of Bcc. One, two or three putative GMD-encoding genes were found in sequenced strains of the *Burkholderia* genus (Fig. S1 panel A). Some Bcc strains contained only the *bceN* homologous gene, such as the *B. multivorans* ATCC 17616. Therefore, we have also compared the EPS production by *B. multivorans* ATCC 17616 and its derivative strain *B. multivorans* (pJFR6) induced with arabinose. Induction of the antisense *bceN* mRNA in *B. multivorans* (pJFR6) strain completely abolished the EPS production (Fig. 4). Taken together, our results strongly suggest that the BceN activity is required for the biosynthesis of cepacian. In fact, in the *B. multivorans*, with a unique gene putatively encoding a protein with GMD activity, no cepacian was detected when an antisense targeting the *bceN* mRNA was expressed. In the case of *B. cepacia* IST408 encoding more than one protein with GMD activity, the inactivation of the *bceN* gene did not led to the abolishment of cepacian production. This is most probably due to the expression of additional gene(s) encoding protein(s) with GMD activity, thus compensating the inactivation of the *bceN* gene.

## Discussion

In this work we report the cloning, purification, biochemical and functional characterization of the *bceN* gene from the opportunistic pathogens *B. cenocepacia* J2315 and *B. cepacia* IST408, which are 97% identical. This last strain is a clinical isolate previously reported as a high producer of cepacian [8], and was used in this study since *B. cenocepacia* J2315 does not produce cepacian due to a frameshift mutation on the *bceB* gene [18].

The *bceN* gene is part of the *bceII* cluster of genes found in all the genomes of *Burkholderia* strains sequenced so far [17]. Amino acid sequence analysis, together with the predicted secondary and tertiary structures of the BceN proteins revealed conserved domains typical of GDP-D-mannose dehydratases (GMD). These proteins belong to the short-chain dehydrogenases/reductases (SDR) superfamily. The SDR protein superfamily has raised special interest due to their molecular evolution, enzymology and biotechnological applications [29].

BceN homologue proteins are involved in the synthesis of the rare deoxysugar nucleotide GDP-D-rhamnose, as is the case of the *P. aeruginosa* GMD [5], the *H. pylori* HP0044 [39], or the *E. coli* GMD [30]. GDP-D-rhamnose biosynthesis involves two steps [5], [31]. The first step is the elimination of a water molecule from GDP-D-mannose by the GMD enzyme activity, yielding GDP-4-keto-6-deoxy-D-mannose. This labile intermediary is further converted into GDP-D-rhamnose by the enzyme activity GDP-D-mannose reductase (RMD) that catalyses the reduction of the 4-keto group, being the NAD(P)H cofactor the electron donor.

The *B. cenocepacia* J2315 BceN protein enzyme activity was investigated based on NMR analysis. This technique was used previously by others to identify the products of the reaction catalyzed by the GMD and RMD enzymes of *P. aeruginosa* [5], as well as of other enzyme activities [27]. NMR spectra obtained for the reaction products of the *B. cenocepacia* J2315 BceN protein indicate that this protein is monofunctional. This is not the case of the *P. aeruginosa* protein with GMD activity, which is bifunctional and can catalyze the two reactions necessary to convert GDP-D-mannose into GDP-D-rhamnose [5]. Contrasting with the *P. aeruginosa* GMD protein, the GMD proteins from *H. pylori* and *E. coli* were both found to catalyse only the 4,6 dehydratase reaction, thus yielding only the intermediate GDP-4-keto-6-deoxy-D-mannose [30], [39]. In the case of *B. cenocepacia* J2315, the conversion of the intermediate into the final product GDP-D-rhamnose is most probably performed by the product of the *bceM* gene, previously predicted to encode a GDP-6-deoxy-D-lyxo-4-hexulose reductase. *bceM* is also located in *bceII* cluster, downstream *bceN* [17]. However, the *bceM* gene has not yet been functionally characterized.

The active form of the *B. cenocepacia* BceN was found to be a tetramer, like the GMD proteins from *P. aeruginosa* and *Helicobacter pylori* [31], [39]. However, other proteins with GMD activity have been described as dimers, like the *K. pneumoniae*, *E. coli*, and the Human GMDs [30], [40], [41].

The addition of exogenous NAD^+^ or NADP^+^ did not affect the rate of the reaction catalysed by *B. cenocepacia* J2315. Since the dehydration step requires an electron acceptor [34], it is quite possible that the electron acceptor co-purified with the protein, as previously described for the GMD enzyme of *E. coli* GMD and *P. aeruginosa* GMD [5], [42].

The 6×His-tagged BceN exhibited a non-Michaelis-Menten kinetics, with a typical substrate inhibition model and a cooperative binding to the substrate. The kinetic parameters for 6×His-tagged BceN are comparable with those of other GMD enzymes previously described, such as the *P. aeruginosa* GMD, the *H. pylori* HP0044 and the *E. coli* GMD [5], [30], [39]. A strong inhibition by the substrate GDP-D-mannose was found for the 6×His-tagged BceN enzyme. It is possible that this strong inhibition has evolved to tightly regulate the levels of GDP-D-mannose, preventing an excessive consumption of this substrate required for other cellular processes besides cepacian biosynthesis.

In the *P. aeruginosa* alginate pathway, the step catalysed by the GDP-mannose dehydrogenase is essential for the control of the biosynthetic pathway [1]. In contrast, mutation of the genes encoding enzyme activities involved in the formation of the sugar nucleotides required for the biosynthesis of the exopolyssacharide cepacian did not abolish polysaccharide production [20], [43]. This has been attributed to the occurrence in their genomes of multiple genes encoding proteins with similar function and that are required for other biosynthetic pathways, such as the formation of cell wall polysaccharides and lipopolysaccharides. Zlosnik and Speert [13] reported that the only genes of the cepacian biosynthetic operon that were differentially expressed between a mucoid and a nonmucoid variant of *B. cenocepacia* isolated from a CF patient with chronic infection were the *bceF* and *bceG* genes, encoding a tyrosine autokinase and a glycosyltransferase, respectively. This is in good agreement with previous work, in which a *bceF* mutant from *B. cepacia* IST408 was not able to produce EPS, exhibiting a reduced virulence in a X-CGD mice model when compared with the wild-type strain [15].

Besides the functional analysis of the *bceN* gene, results from this work reinforce previous observations that the control of cepacian biosynthesis does not occur at the sugar nucleotide level, when additional genes encoding similar functions are encoded within the genomes, as are the BceA, BceC and BceN [20], [43]. However, when a single gene is present in the genomes, as in the case of the GMD activity encoded by *bceN* in the *B. multivorans* ATCC17616, mutation in genes encoding protein functions at the sugar nucleotide level can abolish cepacian biosynthesis.

## Supporting Information

Figure S1 ***Burkholderia*** **strains encode 1 to 3 proteins with GMD activity.** (**A**) Genomic context of the *bceN* gene orthologues (represented as solid black arrows) in sequenced genomes of strains of the *Burkholderia* genus. (**B**) Unrooted phylogenetic tree for the *B. cepacia* IST408 BceN and the putative BceN orthologues from *B. lata* sp. 383 (B383), *B. cenocepacia* J2315 (BcenJ), *B. cenocepacia* AU1054, *B. cenocepacia* HI2424 and *B. cenocepacia* MC0-3 (Bcen), *B. cenocepacia* PC184 (BcenP), *B. ambifaria* AMMD (Bamb), *B. dolosa* AUO158 (Bdol), *B. vietnamiensis* G4 (Bviet), *B. thailandensis* E264 (Bthai), *B. multivorans* ATCC17616 (Bmul), *B. mallei* ATCC23344 (Bmal) and *B. pseudomallei* K96243 (Bpseud). The phylogenetic tree was constructed based on the alignment of amino acid sequences with CLUSTAL X2 using the neighbor-joining method with a minimum of 100 bootstraps. Clade A includes putative GMDs located in gene clusters similar to *bce-II* of chromosome 2 that is involved in Cepacian biosynthesis [17]. Clade B includes the *Burkholderia* putative GMD homologies located on chromosome 1, and the surrounding genes, arranged in clusters most probably involved in LPS biosynthesis. The *B. vietnamiensis* G4 BceN ortologue is not included in clade A or B, and is the only GMD homologue from a *Burkholderia* strain encoded on chromosome 3. Genes: a, b – ABC transporters; c, j, m, o – glycosiltransferases; d – type II mannose-6-phosphate isomerase; e – transposase; f – GDP-D-mannose 4,6-desydratase; g, k – NAD-dependent epimerases/dehydratases; h – methyltransferase FkbM; i – hypothetical protein; l – methyltransferase type 11; n – *wcbA*; p – *wcbC*; q – *bexA*; r – *bexB*; s – *wcbD*; t – transferase hexapeptide repeat containing protein; u – polysaccharide biosynthesis protein; v – exopolysacharide transport protein; x – tyrosine phosphatase; w – polysaccharide export protein; y – sugar transferase.
(DOC)

Table S1 **Bacterial strains and plasmids used in this work.**
(DOC)

Table S2 **^13^C and ^1^H NMR data for GDP-D-mannose (A), GDP-4-keto-6-deoxy-D-mannose (B), and the gem-diol form of compound B (C).** Experiment with 5 mM GDP-D-mannose, 14 hours of incubation with 90 µg BceN protein and 10 mM MgCl_2_ in H_2_O 90%/D_2_O 10% buffered saline solution. Resonances were referenced to an internal acetone standard at δ_H_ = 2.225 p.p.m and δ_C_ = 30.89 p.p.m. For compounds A, B and C, the coupling constants (J_HH_) for the anomeric proton were 2.08 Hz, 1.56 Hz and 1.56 Hz, respectively. * Low resolution or signals obscured by glycerol peaks.
(DOC)
