# Supplementary material for: Biochemical and Functional Studies on the Burkholderia cepacia Complex bceN Gene, Encoding a GDP-D-Mannose 4,6-Dehydratase
Source: PLoS One. 2013 Feb 27;8(2):e56902. doi: 10.1371/journal.pone.0056902 (PMC3584063; doi:10.1371/journal.pone.0056902)
Supplement: Figure S1 — Burkholderia strains encode 1 to 3 proteins with GMD activity. (A) Genomic context of the bceN gene orthologues (represented as solid black arrows) in sequenced genomes of strains of the Burkholderia genus. (B) Unrooted phylogenetic tree for the B. cepacia IST408 BceN and the putative BceN orthologues from B. lata sp. 383 (B383), B. cenocepacia J2315 (BcenJ), B. cenocepacia AU1054, B. cenocepacia HI2424 and B. cenocepacia MC0-3 (Bcen), B. cenocepacia PC184 (BcenP), B. ambifaria AMMD (Bamb), B. dolosa AUO158 (Bdol), B. vietnamiensis G4 (Bviet), B. thailandensis E264 (Bthai), B. multivorans ATCC17616 (Bmul), B. mallei ATCC23344 (Bmal) and B. pseudomallei K96243 (Bpseud). The phylogenetic tree was constructed based on the alignment of amino acid sequences with CLUSTAL X2 using the neighbor-joining method with a minimum of 100 bootstraps. Clade A includes putative GMDs located in gene clusters similar to bce-II of chromosome 2 that is involved in Cepacian biosynthesis [17]. Clade B includes the Burkholderia putative GMD homologies located on chromosome 1, and the surrounding genes, arranged in clusters most probably involved in LPS biosynthesis. The B. vietnamiensis G4 BceN ortologue is not included in clade A or B, and is the only GMD homologue from a Burkholderia strain encoded on chromosome 3. Genes: a, b – ABC transporters; c, j, m, o – glycosiltransferases; d – type II mannose-6-phosphate isomerase; e – transposase; f – GDP-D-mannose 4,6-desydratase; g, k – NAD-dependent epimerases/dehydratases; h – methyltransferase FkbM; i – hypothetical protein; l – methyltransferase type 11; n – wcbA; p – wcbC; q – bexA; r – bexB; s – wcbD; t – transferase hexapeptide repeat containing protein; u – polysaccharide biosynthesis protein; v – exopolysacharide transport protein; x – tyrosine phosphatase; w – polysaccharide export protein; y – sugar transferase. (DOC) [file pone.0056902.s001.doc]

**Figure S1**

**B**

**A**

**O**

***d***

***j***

***i***

***f***

**103587**

***b***

**826458**

**-**

**843509**

***f***

***m***

***m***

***m***

***a***

***g***

***i***

***k***

**41286**

**-**

**34480**

***h***

***m***

***o***

***o***

***n***

***p***

***r***

***g***

***o***

***f***

***o***

***q***

***s***

**1130340**

**13453**

**Chromosome**

**2**

***bceM***

***N***

***P***

***Q***

***R***

***S***

***B.***

***cenocepacia***

**HI2424**

***B.***

***cenocepacia***

**AU 1054**

***B.***

***cenocepacia***

**J2315**

***B.***

***cenocepacia***

**MC0**

**-**

**3**

***B.***

***ambifaria***

**MC40**

**-**

**6**

***B.***

***ambifaria***

**AMMD**

***B.***

***multivorans***

**ATCC 17616**

***B.***

***thailandensis***

**E264**

***B.***

***pseudomallei***

**K96243**

***B.***

***mallei***

**ATCC23344**

**850847**

**1630274**

**1111224**

**442971**

**854312**

**151001**

**1717963**

**805233**

**2324036**

**1871482**

**862112**

**1619009**

**1122512**

**431709**

**865660**

**163539**

**1706796**

**816985**

**2312964**

**1860440**

***a***

***k***

***j***

***i***

***i***

***h***

***g***

***f***

***d***

***c***

***b***

***k***

***h***

***g***

***f***

***d***

***e***

***d***

***c***

***b***

***a***

***k***

***g***

***f***

***b***

***a***

***k***

***m***

***g***

***f***

***b***

***a***

**979262**

**433244**

**852119**

**823749**

**1487069**

**994100**

**449445**

**865061**

**849007**

**1504239**

***bceM***

***N***

***O***

***P***

***Q***

***R***

***S***

***bceM***

***N***

***O***

***P***

***Q***

***R***

***S***

***bceM***

***N***

***O***

***P***

***Q***

***R***

***S***

***bceM***

***N***

***O***

***P***

***Q***

***R***

***bceM***

***N***

***O***

***P***

***Q***

***R***

***S***

***bceM***

***N***

***P***

***Q***

***R***

***S***

***bceM***

***N***

***O***

***P***

***Q***

***R***

***S***

***bceM***

***N***

***O***

***P***

***Q***

***R***

***S***

***bceM***

***N***

***O***

***P***

***Q***

***R***

***m***

***m***

***m***

***i***

***m***

***i***

***m***

***l***

**Chromosome**

**1**

***B. lata***

**383**

**2427820**

**2416355**

***bceM***

***N***

***O***

***P***

***Q***

***R***

***S***

***B.***

***vietnamiensis***

**G4**

**1310148**

**1321735**

***bceM***

***N***

***O***

***Q***

***R***

***S***

**Chromosome**

**3**

**1143860**

***f***

***g***

***m***

***i***

***t***

***u***

***d***

***v***

***x***

***w***

***y***

***bceM***

***N***

***S***

***B.***

***cenocepacia***

**PC184**

**2161**

***B. dolosa***

**AU0158**

***bceM***

***N***

***O***

***P***

***Q***

**12665**

**19979**

***a***

***m***

***g***

***b***

***f***

***c***

***d***

***k***

**125857**

***m***

***d***

**44064**

**13523**

**23091**

**-**

**17017**

**1kb**

***j***

***d***

***j***

***i***

***f***

**103587**

***b***

**826458**

**-**

**843509**

***f***

***m***

***m***

***m***

***a***

***g***

***i***

***k***

**41286**

**-**

**34480**

***h***

***m***

***o***

***o***

***n***

***p***

***r***

***g***

***o***

***f***

***o***

***q***

***s***

**1130340**

**13453**

***bceM***

***N***

***P***

***Q***

***R***

***S***

***B.***

***cenocepacia***

**HI2424**

***B.***

***cenocepacia***

**AU 1054**

***B.***

***cenocepacia***

**J2315**

***B.***

***cenocepacia***

**MC0**

**-**

**3**

***B.***

***ambifaria***

**MC40**

**-**

**6**

***B.***

***ambifaria***

**AMMD**

***B.***

***multivorans***

**ATCC 17616**

***B.***

***thailandensis***

**E264**

***B.***

***pseudomallei***

**K96243**

***B.***

***mallei***

**ATCC23344**

**850847**

**1630274**

**1111224**

**442971**

**854312**

**151001**

**1717963**

**805233**

**2324036**

**1871482**

**862112**

**1619009**

**1122512**

**431709**

**865660**

**163539**

**1706796**

**816985**

**2312964**

**1860440**

***a***

***k***

***j***

***i***

***i***

***h***

***g***

***f***

***d***

***c***

***b***

***k***

***h***

***g***

***f***

***d***

***e***

***d***

***c***

***b***

***a***

***k***

***g***

***f***

***b***

***a***

***k***

***m***

***g***

***f***

***b***

***a***

**979262**

**433244**

**852119**

**823749**

**1487069**

**994100**

**449445**

**865061**

**849007**

**1504239**

***bceM***

***N***

***O***

***P***

***Q***

***R***

***S***

***bceM***

***N***

***O***

***P***

***Q***

***R***

***S***

***bceM***

***N***

***O***

***P***

***Q***

***R***

***S***

***bceM***

***N***

***O***

***P***

***Q***

***R***

***bceM***

***N***

***O***

***P***

***Q***

***R***

***S***

***bceM***

***N***

***P***

***Q***

***R***

***S***

***bceM***

***N***

***O***

***P***

***Q***

***R***

***S***

***bceM***

***N***

***O***

***P***

***Q***

***R***

***S***

***bceM***

***N***

***O***

***P***

***Q***

***R***

***m***

***m***

***m***

***i***

***m***

***i***

***m***

***l***

***B. lata***

**383**

**2427820**

**2416355**

***bceM***

***N***

***O***

***P***

***Q***

***R***

***S***

***B.***

***vietnamiensis***

**G4**

**1310148**

**1321735**

***bceM***

***N***

***O***

***Q***

***R***

***S***

**1143860**

***f***

***g***

***m***

***i***

***t***

***u***

***d***

***v***

***x***

***w***

***y***

***bceM***

***N***

***S***

***B.***

***cenocepacia***

**PC184**

**2161**

***B. dolosa***

**AU0158**

***bceM***

***N***

***O***

***P***

***Q***

**12665**

**19979**

***a***

***m***

***g***

***b***

***f***

***c***

***d***

***k***

**125857**

***m***

***d***

**44064**

**13523**

**23091**

**-**

**17017**

**1kb**

***j***

***O P Q R***

***O***

***S***

***P***

**A**

**O**

***d***

***j***

***i***

***f***

**103587**

***b***

**826458**

**-**

**843509**

***f***

***m***

***m***

***m***

***a***

***g***

***i***

***k***

**41286**

**-**

**34480**

***h***

***m***

***o***

***o***

***n***

***p***

***r***

***g***

***o***

***f***

***o***

***q***

***s***

**1130340**

**13453**

**Chromosome**

**2**

***bceM***

***N***

***P***

***Q***

***R***

***S***

***B.***

***cenocepacia***

**HI2424**

***B.***

***cenocepacia***

**AU 1054**

***B.***

***cenocepacia***

**J2315**

***B.***

***cenocepacia***

**MC0**

**-**

**3**

***B.***

***ambifaria***

**MC40**

**-**

**6**

***B.***

***ambifaria***

**AMMD**

***B.***

***multivorans***

**ATCC 17616**

***B.***

***thailandensis***

**E264**

***B.***

***pseudomallei***

**K96243**

***B.***

***mallei***

**ATCC23344**

**850847**

**1630274**

**1111224**

**442971**

**854312**

**151001**

**1717963**

**805233**

**2324036**

**1871482**

**862112**

**1619009**

**1122512**

**431709**

**865660**

**163539**

**1706796**

**816985**

**2312964**

**1860440**

***a***

***k***

***j***

***i***

***i***

***h***

***g***

***f***

***d***

***c***

***b***

***k***

***h***

***g***

***f***

***d***

***e***

***d***

***c***

***b***

***a***

***k***

***g***

***f***

***b***

***a***

***k***

***m***

***g***

***f***

***b***

***a***

***d***

***j***

***i***

***f***

**103587**

***b***

**826458**

**-**

**843509**

***f***

***m***

***m***

***m***

***a***

***g***

***i***

***k***

**41286**

**-**

**34480**

***h***

***m***

***o***

***o***

***n***

***p***

***r***

***g***

***o***

***f***

***o***

***q***

***s***

**1130340**

**13453**

**Chromosome**

**2**

***bceM***

***N***

***P***

***Q***

***R***

***S***

***B.***

***cenocepacia***

**HI2424**

***B.***

***cenocepacia***

**AU 1054**

***B.***

***cenocepacia***

**J2315**

***B.***

***cenocepacia***

**MC0**

**-**

**3**

***B.***

***ambifaria***

**MC40**

**-**

**6**

***B.***

***ambifaria***

**AMMD**

***B.***

***multivorans***

**ATCC 17616**

***B.***

***thailandensis***

**E264**

***B.***

***pseudomallei***

**K96243**

***B.***

***mallei***

**ATCC23344**

**850847**

**1630274**

**1111224**

**442971**

**854312**

**151001**

**1717963**

**805233**

**2324036**

**1871482**

**862112**

**1619009**

**1122512**

**431709**

**865660**

**163539**

**1706796**

**816985**

**2312964**

**1860440**

***a***

***k***

***j***

***i***

***i***

***h***

***g***

***f***

***d***

***c***

***b***

***k***

***h***

***g***

***f***

***d***

***e***

***d***

***c***

***b***

***a***

***k***

***g***

***f***

***b***

***a***

***k***

***m***

***g***

***f***

***b***

***a***

**979262**

**433244**

**852119**

**823749**

**1487069**

**994100**

**449445**

**865061**

**849007**

**1504239**

***bceM***

***N***

***O***

***P***

***Q***

***R***

***S***

***bceM***

***N***

***O***

***P***

***Q***

***R***

***S***

***bceM***

***N***

***O***

***P***

***Q***

***R***

***S***

***bceM***

***N***

***O***

***P***

***Q***

***R***

***bceM***

***N***

***O***

***P***

***Q***

***R***

***S***

**979262**

**433244**

**852119**

**823749**

**1487069**

**994100**

**449445**

**865061**

**849007**

**1504239**

***bceM***

***N***

***O***

***P***

***Q***

***R***

***S***

***bceM***

***N***

***O***

***P***

***Q***

***R***

***S***

***bceM***

***N***

***O***

***P***

***Q***

***R***

***S***

***bceM***

***N***

***O***

***P***

***Q***

***R***

***bceM***

***N***

***O***

***P***

***Q***

***R***

***S***

***bceM***

***N***

***P***

***Q***

***R***

***S***

***bceM***

***N***

***O***

***P***

***Q***

***R***

***S***

***bceM***

***N***

***O***

***P***

***Q***

***R***

***S***

***bceM***

***N***

***O***

***P***

***Q***

***R***

***m***

***m***

***m***

***i***

***m***

***i***

***m***

***l***

**Chromosome**

**1**

***B. lata***

**383**

**2427820**

**2416355**

***bceM***

***N***

***O***

***P***

***Q***

***R***

***S***

***B.***

***vietnamiensis***

**G4**

**1310148**

**1321735**

***bceM***

***N***

***O***

***Q***

***R***

***S***

**Chromosome**

**3**

**1143860**

***f***

***g***

***m***

***i***

***t***

***u***

***d***

***v***

***x***

***w***

***y***

***bceM***

***N***

***S***

***B.***

***cenocepacia***

**PC184**

**2161**

***bceM***

***N***

***P***

***Q***

***R***

***S***

***bceM***

***N***

***O***

***P***

***Q***

***R***

***S***

***bceM***

***N***

***O***

***P***

***Q***

***R***

***S***

***bceM***

***N***

***O***

***P***

***Q***

***R***

***m***

***m***

***m***

***i***

***m***

***i***

***m***

***l***

**Chromosome**

**1**

***B. lata***

**383**

**2427820**

**2416355**

***bceM***

***N***

***O***

***P***

***Q***

***R***

***S***

***B.***

***vietnamiensis***

**G4**

**1310148**

**1321735**

***bceM***

***N***

***O***

***Q***

***R***

***S***

**Chromosome**

**3**

**1143860**

***f***

***g***

***m***

***i***

***t***

***u***

***d***

***v***

***x***

***w***

***y***

***bceM***

***N***

***S***

***B.***

***cenocepacia***

**PC184**

**2161**

***B. dolosa***

**AU0158**

***bceM***

***N***

***O***

***P***

***Q***

**12665**

**19979**

***a***

***m***

***g***

***b***

***f***

***c***

***d***

***k***

**125857**

***m***

***d***

**44064**

**13523**

**23091**

**-**

**17017**

**1kb**

***j***

***d***

***j***

***i***

***f***

**103587**

***b***

**826458**

**-**

**843509**

***f***

***m***

***m***

***m***

***a***

***g***

***i***

***k***

**41286**

**-**

**34480**

***h***

***m***

***o***

***o***

***n***

***p***

***r***

***g***

***o***

***f***

***o***

***q***

***s***

**1130340**

**13453**

***bceM***

***N***

***P***

***Q***

***R***

***S***

***B.***

***cenocepacia***

**HI2424**

***B.***

***cenocepacia***

**AU 1054**

***B.***

***cenocepacia***

**J2315**

***B.***

***cenocepacia***

**MC0**

**-**

**3**

***B.***

***ambifaria***

**MC40**

**-**

**6**

***B.***

***ambifaria***

**AMMD**

***B.***

***multivorans***

**ATCC 17616**

***B.***

***thailandensis***

**E264**

***B.***

***pseudomallei***

**K96243**

***B.***

***mallei***

**ATCC23344**

**850847**

**1630274**

**1111224**

**442971**

**854312**

**151001**

**1717963**

**805233**

**2324036**

**1871482**

**862112**

**1619009**

**1122512**

**431709**

**865660**

**163539**

**1706796**

**816985**

**2312964**

**1860440**

***a***

***k***

***j***

***i***

***i***

***h***

***g***

***f***

***d***

***c***

***b***

***k***

***h***

***g***

***f***

***d***

***e***

***d***

***c***

***b***

***a***

***k***

***g***

***f***

***b***

***a***

***k***

***m***

***g***

***f***

***b***

***a***

**979262**

**433244**

**852119**

**823749**

**1487069**

**994100**

**449445**

**865061**

**849007**

**1504239**

***bceM***

***N***

***O***

**AMMD**

***B.***

***multivorans***

**ATCC 17616**

***B.***

***thailandensis***

**E264**

***B.***

***pseudomallei***

**K96243**

***B.***

***mallei***

**ATCC23344**

**850847**

**1630274**

**1111224**

**442971**

**854312**

**151001**

**1717963**

**805233**

**2324036**

**1871482**

**862112**

**1619009**

**1122512**

**431709**

**865660**

**163539**

**1706796**

**816985**

**2312964**

**1860440**

***a***

***k***

***j***

***i***

***i***

***h***

***g***

***f***

***d***

***c***

***b***

***k***

***h***

***g***

***f***

***d***

***e***

***d***

***c***

***b***

***a***

***k***

***g***

***f***

***b***

***a***

***k***

***m***

***g***

***f***

***b***

***a***

**979262**

**433244**

**852119**

**823749**

**1487069**

**994100**

**449445**

**865061**

**849007**

**1504239**

***bceM***

***N***

***O***

***P***

***Q***

***R***

***S***

***bceM***

***N***

***O***

***P***

***Q***

***R***

***S***

***bceM***

***N***

***O***

***P***

***Q***

***R***

***S***

***bceM***

***N***

***O***

***P***

***Q***

***R***

***bceM***

***N***

***O***

***P***

***Q***

***R***

***S***

***bceM***

***N***

***P***

***Q***

***R***

***S***

***bceM***

***N***

***O***

***P***

***Q***

***R***

***S***

***bceM***

***N***

***O***

***P***

***Q***

***R***

***S***

***bceM***

***N***

***O***

***P***

***Q***

***R***

***m***

***m***

***m***

***i***

***m***

***i***

***m***

***l***

***B. lata***

**383**

**2427820**

**2416355**

***bceM***

***N***

***O***

***P***

***Q***

***R***

***S***

***B.***

***vietnamiensis***

**G4**

**1310148**

**1321735**

***bceM***

***N***

***O***

***Q***

***R***

***S***

**1143860**

***f***

***g***

***m***

***i***

***t***

***u***

***d***

***v***

***x***

***w***

***y***

***bceM***

***N***

***S***

***B.***

***cenocepacia***

**PC184**

**2161**

***B. dolosa***

**AU0158**

***bceM***

***N***

***O***

***P***

***Q***

**12665**

**19979**

***a***

***m***

***g***

***b***

***f***

***c***

***d***

***k***

**125857**

***m***

***d***

**44064**

**13523**

**23091**

**-**

**17017**

**1kb**

***j***

***O P Q R***

***O***

***S***

***P***

**0.01**
