# Supplementary material for: Biochemical and Functional Studies on the Burkholderia cepacia Complex bceN Gene, Encoding a GDP-D-Mannose 4,6-Dehydratase
Source: PLoS One. 2013 Feb 27;8(2):e56902. doi: 10.1371/journal.pone.0056902 (PMC3584063; doi:10.1371/journal.pone.0056902)
Supplement: Table S1 — Bacterial strains and plasmids used in this work. (DOC) [file pone.0056902.s002.doc]

**Table S**1. Bacterial strains and plasmids used in this work.

| **Strain or plasmid** | **Genotype or description** | **Reference or source** |
| --- | --- | --- |
| **Strains** |  |  |
| *B. cenocepacia* J2315 | Cystic fibrosis clinical isolate (UK); ET12 lineage reference strain | 45 |
| *B. cepacia* IST408 | Cystic fibrosis clinical isolate (Hospital Santa Maria, Portugal) | 8 |
| *B. multivorans* ATCC17616 | Soil isolate (Berkeley, California, USA) | 6 |
| *B. cepacia* JRF1 | *B. cepacia* IST408 derivative with the *bceN* gene interrupted with a trimethoprim cassette | This study |
| *B. multivorans* (pJFR6) | *B. multivorans* ATCC17616 transformed with pJFR6 | This study |
| *Escherichia coli* DH5α | F^-^ *endA1 glnV44 thi-1 recA1 relA1 gyrA96 deoR nupG* Φ80d*lacZ*ΔM15 Δ(*lacZYA-argF*)*U169*, *hsdR17*(r_K_^-^ m_K_^+^), λ^–^ | Invitrogen |
| *E. coli* BL21 (DE3) | F^-^ *ompT* *hsd*S_B_ (r_B_^-^m_B_^-^) *dcm gal* λ(DE3). | Stratagene |
| *E. coli* SSC110 | *dam dcm endA1 supE44 hsdR17 thi leu rpsL1 lacY galK galT ara tonA thr* Δ(*lac-proAB*)*/*F' [*traD36 proAB*^+^ *lacI*^q^ *lacZ*ΔM15]. | Stratagene |
| *E. coli* HB101 | *thi-1 hsd*S20(r_B_^-^, m_B_^-^) *sup*E44 *rec*A13 *ara*-14 *leu*B6 *pro*A2 *lac*Y1 *gal*K 2*rps*L20 (Str^r^) *xyl*-5 *mti*-1. | Promega |
|  |  |  |
| **Plasmids** |  |  |
| pDrive | Cloning vector, Km^r^, Ap^r^ | Qiagen |
| pUC-Tp | pUC19 with the insertion of trimethoprim cassette, Tp^r^ and Ap^r^ | 20 |
| pET23a+ | Cloning/expression vector, Ap^r^ | Novagen |
| pMLBAD | pBBR1 *ori*, *araC*/P_BAD_, *mob,* Tp^r^ | 46 |
| pRK2013 | Mobilizing vector, ColE1 tra (RK2)^+^, Km^r^ | 47 |
| pACM2 | pDrive containing the 2272 bp KpnI/HindIII fragment containing the *bceN* gene and flanking regions from *B. cenocepacia* J2315, Ap^r^, Km^r^ | This study |
| pACM4 | pACM2 with the trimethoprim cassette insertion in the *bceN* gene, Ap^r^, Tp^r^, Km^r^ | This study |
| pJFR2 | pDrive containing the 1061 bp HincII fragment containing the *bceN* gene from *B. cepacia* IST408, Ap^r^ Km^r^ | This study |
| pJFR4 | pET23a+ containing the 1061 bp NheI/HindIII fragment containing the *bceN* gene from *B. cenocepacia* J2315, C-terminal His-tag, Ap^r^ | This study |
| pJFR5 | pDrive containing the 1061 bp XbaI/HindIII fragment from pJFR4 containing the *B. cenocepacia* J2315 *bceN* gene | This study |
| pJFR6 | pMLBAD containing the 1061 bp KpnI/XbaI fragment from pJFR5 with the *bceN* gene from *B. cenocepacia* J2315 cloned in the 3’-5’ direction, thus expressing a *bceN* antisense RNA. | This study |

6 Leitão JH, Sousa SA, Ferreira AS, Ramos CG, Silva IN, et al. (2010) Pathogenicity, virulence factors, and strategies to fight against *Burkholderia cepacia* complex pathogens and related species. Appl Microbiol Biotechnol 87: 31-40.

8 Richau JA, Leitão JH, Correia M, Lito L, Salgado MJet al. (2000) Molecular typing and exopolysaccharide biosynthesis of *Burkholderia cepacia* isolates from a Portuguese cystic fibrosis center. J Clin Microbiol 38: 1651–1655.

45 Govan JR, Brown PH, Maddison J, Doherty CJ, Nelson JW, et al. (1993) Evidence for transmission of *Pseudomonas cepacia* by social contact in cystic fibrosis. Lancet 342:15–19.

46 Lefebre MD, Valvano MA (2002) Construction and evaluation of plasmid vectors optimized for constitutive and regulated gene expression in *Burkholderia cepacia* complex isolates. Appl Environ Microbiol 68: 5956–5964.

47 Figurski DH, Helinski DR (1979) Replication of an origin-containing derivative of plasmid RK2 dependent on a plasmid function provided in trans. Proc Natl Acad Sci USA 76: 1648-1652.
