# Supplementary material for: Biochemical and Functional Studies on the Burkholderia cepacia Complex bceN Gene, Encoding a GDP-D-Mannose 4,6-Dehydratase
Source: PLoS One. 2013 Feb 27;8(2):e56902. doi: 10.1371/journal.pone.0056902 (PMC3584063; doi:10.1371/journal.pone.0056902)
Supplement: Table S2 — 13C and 1H NMR data for GDP-D-mannose (A), GDP-4-keto-6-deoxy-D-mannose (B), and the gem-diol form of compound B (C). Experiment with 5 mM GDP-D-mannose, 14 hours of incubation with 90 µg BceN protein and 10 mM MgCl2 in H2O 90%/D2O 10% buffered saline solution. Resonances were referenced to an internal acetone standard at δH = 2.225 p.p.m and δC = 30.89 p.p.m. For compounds A, B and C, the coupling constants (JHH) for the anomeric proton were 2.08 Hz, 1.56 Hz and 1.56 Hz, respectively. * Low resolution or signals obscured by glycerol peaks. (DOC) [file pone.0056902.s003.doc]

**Table S2** – ^13^C and ^1^H NMR data for GDP-D-mannose (A), GDP-4-keto-6-deoxy-D-mannose (B), and the gem-diol form of compound B (C). Experiment with 5 mM GDP-D-mannose, 14 hours of incubation with 90 μg BceN protein and 10 mM MgCl_2_ in H_2_O 90% / D_2_O 10% buffered saline solution. Resonances were referenced to an internal acetone standard at δ_H_ = 2.225 p.p.m and δ_C_ = 30.89 p.p.m. For compounds A, B and C, the coupling constants (J_HH_) for the anomeric proton were 2.08 Hz, 1.56 Hz and 1.56 Hz, respectively. * Low resolution or signals obscured by glycerol peaks.

| Compound |  | Chemical shifts (p.p.m) | | | | | |
| --- | --- | --- | --- | --- | --- | --- | --- |
|  |  | H1 | H2 | H3 | H4 | H5 | H6 |
| A | ^1^H | 5.51 | 4.05 | 3.92 | * | 3.84 | * |
|  | ^13^C | 97.07 | 70.89 | 70.78 | * | 74.2 | * |
| B | ^1^H | 5.58 | 4.46 | * | - | 4.71 | 1.23 |
|  | ^13^C | 96.28 | 75.80 | * | 208.60 | 72.16 | 13.40 |
| C | ^1^H | 5.45 | * | * | - | * | 1.21 |
|  | ^13^C | 97.08 | * | * | * | * | 11.90 |
